# An Explorative Analysis of the Influence of Landscape Visual Aesthetic Quality on Food Preferences in Italy: A Pilot Study

**DOI:** 10.3390/foods11121779

**Published:** 2022-06-16

**Authors:** Tiziano Tempesta, Daniel Vecchiato

**Affiliations:** Department of Land, Environment, Agriculture and Forestry (TESAF), Università degli Studi di Padova, 35020 Legnaro, PD, Italy; daniel.vecchiato@unipd.it

**Keywords:** landscape, preferences, beverage, juice, liking, willingness to pay

## Abstract

As some previous research has highlighted, landscape characteristics are useful for improving the market share of some food products and the market power of companies in the agrifood sector. The purpose of this study is to verify whether the visual aesthetic quality of the landscape can influence food preferences and the willingness to pay for agrifood products. To this end, the preferences of 64 participants for three types of juice (orange, peach and pear) were analysed through a blind tasting experiment. Each participant tasted three pairs of fruit juices, one for each type of juice. The juices belonging to each pair were the same, but before tasting, the participants were shown two photos portraying the orchards where the fruits were produced, so participants were induced to think that the juices were different. The landscape associated with each pair of photographs had a different visual aesthetic quality (high or low). Participants were asked to provide three measures while tasting the juices: their overall juice assessment using a seven-point hedonic scale, the visual aesthetic quality of the photos on a seven-point Likert scale, and their willingness to pay as a percentage variation of the price that they usually pay to buy fruit juices. According to our results, the mean overall liking score and the mean willingness to pay percentage variation for the juices associated with a preferred landscape was higher and statistically different. Despite the need for further research, our results suggest that landscape acts as a proxy for quality in the evaluation of some food products and that the use of landscape photos could be a valid marketing strategy in agribusiness.

## 1. Introduction

Food is defined in economics as an “experience good”, namely, a good whose quality cannot be fully evaluated before tasting it. In the case of a new food product, such an aspect becomes crucial, especially when a consumer needs to decide whether to buy a specific product. In such circumstances, the consumer relies on some signals to infer its quality before tasting it. Quality signals are usually divided into two broad categories: extrinsic and intrinsic cues [1]. Intrinsic cues relate to the product’s physical characteristics (e.g., colour, turbidity, meat marbling) while extrinsic cues do not have a direct relationship with them (e.g., packaging, country of origin). It is important to underscore that the acquisition of information necessary to infer product quality does not only occur in a conscious way [2]; our brain processes many signals related to product quality in a completely unconscious way, also referring to past experience in the consumption of similar goods.

The purchase decision of a new food product is then based on quality expectations, considerations about the quality and price of other substitute products and the budget constraint imposed by personal income.

Only once the product has been purchased and consumed can the consumer verify its real quality [3]. To do this, various attributes are considered that can in turn be divided into two broad categories: experience and credence attributes [4]. Experience attributes concern the characteristics that can be immediately ascertained through consumption (smell, taste, texture), while for credence attributes, this could happen only over a long period of time (e.g., the effects on health of organic products or unsaturated fat content). In other cases, it may never happen. The perception of quality does not depend only on the product to be purchased but can be significantly influenced by the purchase context and emotional state of the consumer [5,6,7,8,9,10,11], as well as by some information relating, for example, to the place of production or the food production technologies [12]. Only once the product has been tasted will the buyer have the opportunity to determine whether it conformed to his/her expectations considering the extrinsic and intrinsic cues. If the expectations are confirmed, it is possible that the good will be purchased again in the future; otherwise (disconfirmation), the product will no longer be purchased.

The factors that can influence consumer preferences are numerous and in some ways difficult to identify in the context of experimental procedures that are necessarily simplified. The role played by extrinsic cues and credence attributes in influencing consumer choice has however been verified by numerous studies that have highlighted how they can influence liking and willingness to pay (WTP) [13,14,15].

In some ways, it can be assumed that the landscape constitutes an extrinsic cue both if the potential buyer has visited the place of production and in the event that the consumer can see an image of the place of production through advertising or on the product packaging. According to the European Landscape Convention, signed in Florence in 2000, “landscape means an area, as perceived by people, whose character is the result of the action and interaction of natural and/or human factors”. Following this definition, a landscape can be considered a visual cue. Many studies highlighted that landscapes could modify the emotional state of people [5,6,7,9,10,11,16,17,18,19]. In this respect, it is possible that when the characteristics of a landscape promote positive emotions, it is perceived as having high visual aesthetic quality and, on the contrary, when it promotes negative emotions, it is perceived as having low visual aesthetic quality. Many studies conducted over the last 50 years have made it possible to identify which factors influence people’s landscape preferences. It has emerged that in rural areas, natural elements (water bodies, woodlands, hedgerows and meadows) tend to improve the landscape’s visual aesthetic quality, whereas some anthropogenic elements worsen it [20,21,22]. Tveit et al. [23], through an analysis of the literature on the evaluation of visual aesthetic landscape quality, selected nine key visual concepts: stewardship, coherence, disturbance, historicity, visual scale, imageability, complexity, naturalness and ephemera. With the exception of disturbance, the other key visual concepts have a positive effect on the aesthetic visual quality that can be defined as a measure of the degree of attraction that a given setting spontaneously exerts on humans.

Food advertising often uses the landscape as an indicator of quality. In order to increase their market power, companies must identify elements that make a product unique and not reproducible by others. From this point of view, especially for certain areas, the landscape is an element that can strongly characterize a product. Contrary to what happens for the packaging and for some organoleptic characteristics, landscapes cannot be reproduced by competitors.

However, to date, only a few studies have tried to analyse the use landscape as a tool for promoting food products and to see if and to what extent it influences food demand. It is possible to classify such studies into two main groups:

1. studies aiming at verifying whether landscape visual aesthetic quality is able to influence food liking [24,25,26]; and
2. studies aiming at verifying whether landscape visual aesthetic quality is able to influence consumer behaviour and WTP [15,27,28,29,30].

The research studies belonging to the first group did not consider the effect of landscape quality on the willingness to pay for food products and on market share. However, in the second group of research studies, the products were not tasted and it was not possible to establish whether there was an interaction between landscape quality, food liking and a propensity to buy the product.

This paper aims to analyse whether visual cues (landscape aesthetic perception in our case) are able to influence the liking and the WTP of a food product. With this aim, a blind tasting experiment was conducted using fruit juices as products. During the tasting, participants tasted the same juices (presented as two different products) and were shown an image of the landscape of the orchards where the fruit used to obtain the juices was produced. More details about the experimental setting used to test our hypotheses will be provided in the Section 2.

## 2. Materials and Methods

### 2.1. Participants

A total of 65 untrained participants were recruited—mostly among classroom students—to analyse the effect of landscape features on the perception of the taste of fruit juices. Among them, 64 were suitable to take part in the experiment, given than one refused to participate for personal reasons (not investigated in order to preserve his/her privacy). We informed the participants about the experiment and the tasting session, providing details about the juices’ components, in order to let the participants understand if the products to be tasted were suitable for them and not causing any allergic reaction. After this introduction, the participants were free to decide whether to take part in the experiment or not. The tastings were done in four distinct sessions. Three sessions were attended only by students. In this case the tastings were carried out in the classroom before the lessons. A session was instead held in the home of one of the authors as part of a specifically organized convivial meeting.

Initially, we informed the participants that research was underway at the Department of Land, Environment, Agriculture and Forestry of the Università degli Studi di Padova (Italy) that was aimed at identifying the effect of the place of production on the taste of fruit juice. The students were not forced to taste the fruit juices nor did they declare their identity in the questionnaire, which is in accordance with the recommendations of the General Data Protection Regulation (GDPR) 2016/679 of the European Union dealing with the protection of personal data.

### 2.2. Stimuli

Considering the aim of the research, two different stimuli were provided to the participants: the first was gustatory and the second was visual. With reference to the first stimulus, participants tasted orange, peach and pear fruit juices that were normal commercial juices of medium price bought in a supermarket. The choice of using fruit juices for the experiment depended on numerous factors. First of all, unlike alcoholic beverages (for example wine or beer), juices can be consumed by practically all people and throughout the day. Only one of the people invited to participate in the experiment was excluded, as he stated that he does not consume fruit juices. It is also a product for which a direct link with the landscape can be easily identified and, furthermore, the orchards can be cultivated in a very different way. Thus, it is possible to relate the juice of a given fruit to different landscapes and to verify the effect that different types of landscape have on the liking of fruit juices.

For the presentation of the juices, we poured them in a 125 mL plastic glass. We did not use transparent plastic glass in order to prevent the possibility that the juice colour might influence the tasting. The juices were served at room temperature.

The tasting was organised in three subsequent sections in which participants tasted a pair of juices of the same fruit labelled A and B, respectively. The juices were the same, but participants were told that they were different because they were obtained from fruit harvested in the two orchards represented in the photos they were looking at. We used six photos as visual stimuli. Photos can have some drawbacks, but, at the same time, they have some advantages. First, photos are simple to use and, second, they can mimic advertising media. In this respect, it should be considered that past studies underlined that people’s reactions to landscape photos may be similar to that ‘in-field’ [31,32]. The images of each pair of tasted juices were shown on the computer screen used to fill in the questionnaire or in high quality photos presented in A4 paper size. 

**Figure 1:**
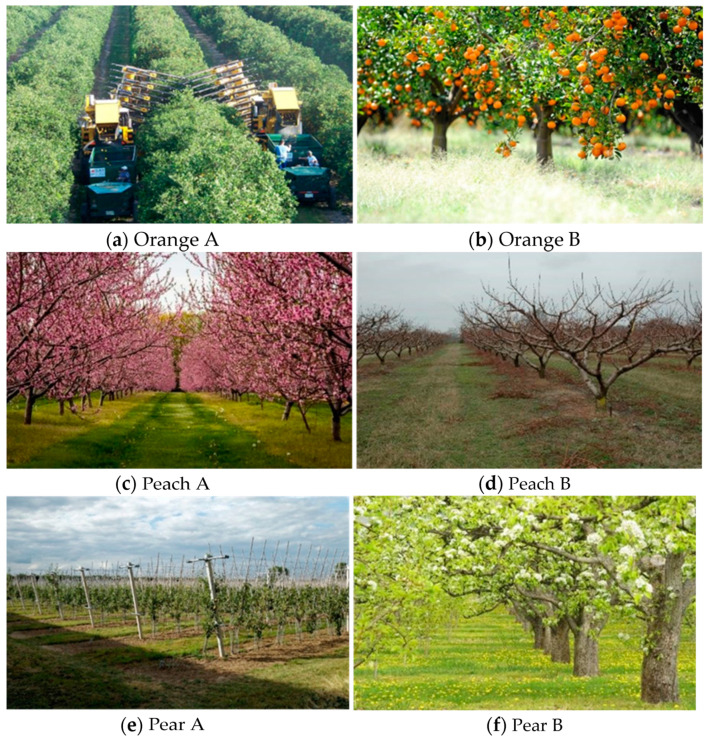
Photos submitted to the participants representing the landscape where the fruit used to produce the tasted juices was obtained.

The pairs of photos relating to each type of orchard were chosen considering some of the nine factors that, according to Tveit et al. [23], can affect the perceptive quality of a landscape (Figure 1 and Table 1). In particular, in each of the pairs of photos we chose a landscape that at least theoretically had a better aesthetic visual quality. Compared to other surveys [25], particularly impactful elements (e.g., modern buildings or factories) or elements of a particular aesthetic value (ancient buildings, historical monuments) were excluded from the photos. In this way, we wanted to verify the effect on the perception of the quality of the tasted product based on landscape elements widespread in the rural areas related to alternative cultivation techniques which can be used to cultivate orchards or various vegetative phases of orchards.

Following the classification of the factors that contribute to improving or worsening the aesthetic preferences of the landscape proposed by Tveit et al. [23], three landscapes of higher aesthetic visual quality (orange B, peach A and pear B) and three of lower aesthetic visual quality (orange A, peach B and pear A) (Figure 1 and Table 1) were identified. It is possible to summarise the experimental design adopted in our study in Table 2.

### 2.3. Sensory Procedure

Participants were requested to taste each pair of juices after observing the pictures representing the landscape of the fruit production environment. First, participants were told to look carefully at the photos of the orchards where the fruits used to produce the juices were harvested. The photos were shown as high quality photos in A4 paper size, and during one session, participants also had them available directly on their computer screens. The participants then tasted the first juice (e.g., Orange A) and expressed their overall liking on a seven-point hedonic scale (where 1 indicates “I don’t like it at all”, 4 indicates neither likes nor dislikes, and 7 indicates “I like it very much”). Finally, they were asked to indicate for each juice whether they would be willing to pay more or less than the mean price usually paid in their place of living for a juice. From a methodological perspective, the WTP measure adopted in this study is grounded in the contingent valuation method [33,34,35] which, despite being developed for the valuation of nonmarket goods, saw several applications in agribusiness marketing [36]. The WTP was measured by asking participants to indicate in a payment card format the percentage variation ranging from −30% to +30%, with an increase of 5% for each option of the scale resulting in 13 values among which participants could choose. They then rinsed their mouth with water and tasted the second fruit juice belonging to the same pair (e.g., Orange B) and made the same assessments indicated for the first tasting.

The same procedure was followed for the other two fruit juices (Peach A and Peach B; Pear A and Pear B). Finally, the participants in the experiment evaluated the aesthetic visual quality of the six landscapes by means of a seven-point Likert scale. The fruit juice bottles were covered with aluminium foil and only an indication of the type of juice (orange, peach or pear) and a letter (A or B) appeared on them. Overall, each participant tasted and evaluated 3 pairs of juices; therefore, 384 evaluations were collected.

As regards the individual characteristics of the participants, age, gender, educational level, field of study and the place of residence (urban or rural) were collected in the questionnaire along with the other ratings.

### 2.4. Statistical Analysis

To analyse the data the following variables were created:

- *JUICE*_*PREF* that assumes a value of 3 for the preferred juice of each pair, 2 when the juices received the same score and 1 for the one not preferred;
- *LAND*_*PREF* that assumes a value of 3 for the preferred landscape of each pair, 2 when the landscapes received the same score and 1 for the one not preferred;
- *WTP*_*PREF* that assumes a value of 3 for the juice of each pair that received the highest WTP, 2 when the juices received the same WTP and 1 for the juice with the lower WTP.

To verify if and to what extent the landscape characteristics affected juice liking and WTP, we first performed both parametric (ANOVA) and nonparametric (Chi-squared) tests.

Then, to understand which factors influenced the preference of one juice over another, we estimated a logistic function by means of the Panel Logistic Regression with the statistical package Stata/MP (version 14.0). As dependent variables of the logistic models, the following dummy variables were used:

- *HIGHEST*_*LIK* that assumes the value 1 for the most liked juice of each pair and 0 in the other cases (equal or lower overall liking)
- *HIGEST*_*WTP* that assumes the value 1 for the juice with the higher WTP of each pair and 0 in the other cases (equal or lower WTP).

## 3. Results

### 3.1. Sample Characteristics

Looking at the sample socioeconomic characteristics (Table A1), women constituted 40.6% of the sample. Regarding the area of residence, most participants resided in rural areas (59.5%). In terms of age distribution, 39.1% were under 25 years, 37.5% were from 25 to 30 years old, and 23.4% were over 30. With reference to the educational level, 42.2% had a university degree, 51.6% had a high school diploma, while the remaining participants had a middle school diploma or lower. In terms of the field of study, 40.6% of the participants had a degree or a diploma in agronomics, 26.6% in the humanities, 15.6% in biological sciences and 12.5% in architecture or engineering.

### 3.2. Landscape Valuation

The participants’ assessment of the landscape aesthetic visual quality was coherent with our classification and our expectations (the rating by landscape is reported in Table A2). With reference to each pair of juices, the landscape that, following Tveit et al. [23] was considered of higher visual aesthetic quality, received the highest score. In the case of the orange juice, landscape B (mean = 5.68, SD = 1.250) was preferred over landscape A (mean = 4.32, SD = 1.06); in the case of the peach juice, landscape A was preferred (mean = 6.48, SD = 0.87) over landscape B (mean = 4.05, SD = 1.73); and finally for the pear juice, landscape B (mean = 6.45, SD = 0.85) was preferred over landscape A (mean = 4.22, SD = 1.69). The means differences were significant for all juices (*p* < 0.001).

### 3.3. Landscape and Juice Liking

Considering the relationship between overall liking and *LAND*_*PREF*, it is possible to observe that *LAND*_*PREF* seems to influence the juice preferences. In fact, the average hedonic score of the overall liking is equal to 4.83 (SD = 1.48) in the case of the not preferred landscapes and to 5.25 (SD = 1.34) in the case of the preferred landscapes (the mean overall liking scores for each juice tasted are reported in Table A3). Between overall liking and landscape visual aesthetic, a positive and significant correlation exists (*r* = 0.23; *p <* 0.001). The hedonic score difference is statistically significant (*p <* 0.05). However, it should be noted that from a food marketing perspective, the mean score attributed to the overall liking for each type of landscape is not the only aspect to be considered. Indeed, it can be assumed that in many ways it is important for the seller to know whether foods produced in a more pleasant landscape are preferred to those obtained in a less pleasant landscape.

To take this aspect into consideration, as previously described, the variable *JUICE*_*PREF* was calculated. As reported in Table 3, the preference between the two tasted juices is significantly influenced by the landscape preference (Chi squared = 46.39, *p* = 0.000). In only 46 cases did the participants prefer the juice (26%) when they did not prefer a landscape more than the other landscape of the same pair; however, they liked the juice more when the landscape of each pair was preferred (in 103 cases, 58.2%).

To better understand which factors influence the probability of juice choice, a logistic regression was estimated where the dependent variable is *HIGHEST*_*LIK* and the independent variable is the landscape aesthetic visual appreciation (Table 4).

As can be seen, the landscape appreciation significantly affects *HIGHEST*_*LIK* and the landscape score seems to have a not negligible effect on juices’ preferences. To analyse the effect of landscape aesthetic and visual quality on the probability that one of the two fruit juices was preferred, we estimated the probability that a juice was chosen when this variable varies (Table 5).

According to the results reported in Table 5, the effect of landscape is always quite relevant. Other things being equal, the probability that one juice is preferred over another increases from 16.09% to 54.16% if the score attributed to the landscape increases from 1 to 7.

### 3.4. Landscape and WTP

As in the case of the overall liking, WTP seems to be influenced by *LAND*_*PREF*. The mean WTP percentage variation is equal to −1.33 (SD = 9.83) in the case of the not preferred landscapes and to +1.64 (SD = 9.73) in the case of the preferred landscapes. The mean difference is statistically significant (*p <* 0.05) (the mean WTPs for each juice tasted are reported in Table A4). There is a positive and significant correlation between the WTP and the landscape visual aesthetic (*r* = 0.18; *p* < 0.001). Moreover, as expected, WTP is positively correlated with the overall liking (*r* = 0.50; *p <* 0.001). With respect to the overall liking, the participants had greater difficulty in expressing their WTP: in 62 pairs out of 194 (nearly 32% of cases), they expressed the same WTP for both tasted products (Table 6). Even for the WTP, it can be seen (Table 6) that *LAND*_*PREF* has a statistically significant influence on the percentage of respondents who were willing to pay more for one of the two tasted juices (Chi squared = 31.74, *p* = 0.000). In the case of the not preferred landscapes, the probability that a higher WTP has been expressed is 22.0%, a percentage which rises to 46.3% in the case of landscapes with a perceived high aesthetic visual quality.

Similarly to *HIGHEST*_*LIK*, a logistic function has been estimated in which the dependent variable is *HIGEST*_*WTP* and the independent variable is landscape aesthetic visual appreciation (Table 7). According to the model results, landscape visual cues are able to influence the market behaviour of people.

Using the model shown in Table 7, it is possible to estimate the effect that the landscape aesthetic visual quality has on the probability that the participants were willing to pay a higher amount of money for one of the juices belonging to the same pair (Table 8). In this case, it can also be seen that the landscape aesthetic preferences seem to have an influence on the participants’ preferences and on their attitude toward the beverage under analysis. In fact, the probability that participants were willing to pay more for one juice increases from 11.33% to 45.76% if the score attributed to the landscape increases from 1 to 7.

## 4. Discussion

According to our results, the landscape aesthetic visual quality seems to be able to influence consumers’ food choices and preferences. A similar effect was also found for the willingness to buy the product. When foods had similar tastiness, landscape quality significantly affected the preferences of the participants. This is of particular importance given that the juices tasted were commercial products of medium-low quality.

Although it is difficult to make a direct comparison with other studies, it should nevertheless be recalled that in this research, the elements of differentiation of the landscape certainly had less perceptual relevance than in other studies. For example, Tempesta et al. [25] found that the landscapes that most influenced wine liking were characterized by the presence of Venetian villas of high architectural quality or vineyards surrounded by natural elements (hedges).

In the research presented by Tomasi et al. [26], the landscapes of high aesthetic visual quality consisted of vineyards located on hills and surrounded by greenery, while those of lower quality were modern vineyards in flat areas. With reference to the classification proposed by Tveit et al. [23], landscapes of high aesthetic visual quality had a high degree of historicity, naturalness and imageability in the work of Tempesta et al. [25], while in that of Tomasi et al. [26] they were characterized by panoramic views (visual scale) and a high degree of naturalness due to the presence of small woods among the vineyards.

Therefore, our results seem to confirm the findings of previous research that analysed the effect of landscape features on liking [24,25,26].

With regard to the effect on WTP, there are no studies in the literature directly comparable with ours. However, the effect of landscape aesthetic visual quality in ensuring a greater WTP was lower than what emerged with regard to tastiness. In this respect, it is important to remember that the purchase of a product depends not only on its perceived quality but also on a comparison with the quality and price of the alternatives. The decision-making process underlying the purchase is, therefore, in part different from that relating to the formation of preferences for food and is probably less influenced by emotions.

It is not easy to understand why the landscape aesthetic visual quality might have influenced the perception of the taste of fruit juices. The landscape in which a product is grown may have no direct relationship with the organoleptic characteristics of food or with the production techniques used to obtain it. However, it should be noted that some extrinsic cues and credence attributes are able to influence both liking and WTP [13,14].

For example, numerous studies have shown that people are willing to pay a premium price for food produced in their country or region of residence [37,38,39]. Others have shown that information about place of production changes the liking of food [40,41,42]. It should be noted that, as in the case of landscape, there is not a univocal relationship between the country of origin and the characteristics of the product. Other authors have pointed out that food preferences can also be influenced by extrinsic characteristics such as packaging [43,44,45] or the context in which tasting takes place [46].

Ultimately, it can be deduced that food preferences depend on a plurality of factors involving various senses, among which sight plays an important role according to Schifferstein and Spence [47]. In this respect, Becker et al. [43], p. 18 stated that “people intuitively make connections between different sensory domains, a phenomenon referred to as cross-modal correspondence”. According to Krishna et al. [48], the purchase of a product is the result of a multisensory customer experience.

A first element that can help explain the effect of landscape aesthetic visual quality on food preferences is the link that exists between emotions and consumption. Environmental stimuli can elicit emotional responses that modify people’s mood and, hence, cause an increase or decrease in the amount of food consumed [16]. Some research has shown that emotional states affect food intake and food preferences [5,6,7] and that visual stimuli (photographs and video clips) affect mood [17,18]. Thus, a possible explanation of the effect of landscape on liking is the fact that the landscape is able to change people’s mood. Some recent studies using virtual reality experiments have highlighted that the emotions associated with food consumption can change depending on the environment in which the food is consumed [9,10,11,19]. A close relationship between liking, emotions and place of consumption has also been highlighted by Xu et al. [19] and Samant and Seo [49].

According to Ulrich [50], landscape preferences have an emotional basis, and many studies have shown, using various approaches, that natural landscapes are able to reduce people’s stress while urban settings are unable to exert any positive effect [51,52,53]; furthermore, more pleasant landscapes favour positive emotions in people [54]. There is also a positive relationship between landscape preferences, restorativeness and emotions aroused by viewing different landscapes [54,55,56,57].

It can therefore be assumed that the respondents unconsciously associated positive emotions with their favorite landscapes and that this association influenced the preferences expressed among the fruit juice pairs evaluated. The presence of such a relationship could be of particular importance, especially in the case of tourists who may have the opportunity to see the landscape where the food is produced; it may also apply to those who make purchases in other contexts since it has been proven that exposure to images that reproduce pleasant landscapes can change the emotional state of consumers [17].

Another possible explanation may be the halo effect. The term “halo effect” was first coined by the psychologist Thorndike [58] and, according to Apaolaza et al. [59], it refers “to a perceptual bias in which one salient attribute determines the overall impression of a person or an object, affecting the perception of other conceptually distinct and independent attributes”. Many studies have demonstrated that the halo effect influences consumers’ preferences for different kinds of food and different signals or quality (e.g., organic label, fair trade, traditional vs. industrial product) [12,59,60,61]. In some ways, it can be supposed that landscape characteristics are used unconsciously to infer at least in part the overall quality of the product. Obviously, the effect of landscape in this case would be less than that of the credence attributes which also influence the quality of the product.

## 5. Conclusions

In conclusion, the landscape visual quality seems to play a not negligible role in favouring one product over another. A similar result also emerged with regard to the willingness to pay a higher price for one product rather than for another.

In evaluating these results, however, the reader should recognise that our study has some limitations. The first is related to the small sample size considered and to the sample selection: the participants in our trials were mainly students. Two other limitations refer to the experiment. In fact, the trials were not randomised and the conditions of the place where the participants tasted the juices were partly different and not fully comparable. Finally, it should be noted that the use of photos might present some limitations in verifying the relationship between liking and landscape quality. In particular, photos may only be a poor proxy of a real in-field experience; thus, they are not completely useful for analysing the effect of visiting a given landscape on food liking. The emotions aroused by direct contact with a landscape may be much more intense than those that felt by looking at a photo. Considering that organizing experiments directly in the food production area can be very expensive, the use of an immersive virtual reality environment could be an interesting alternative for improving the reliability of the results compared to those obtained using photos [9,10,11,62,63].

However, it should be noted that the type of media used to analyse the effect of landscape visual quality on liking essentially depends on the purpose of the study. If the landscape is used as part of an advertising campaign, the research must necessarily be based on photos or videos. In contrast, if the intention is to analyse the effect of a visit, it may be more appropriate to use tools such as immersive virtual reality that allow a better simulation of the relationship that can be established between the landscape and food liking in a given setting [64].

Despite these limitations, our study highlights the possibility that landscape characteristics might increase the market share of a product and the market power of firms. The use of images of the place of production can, therefore, be useful in organizing advertising campaigns for food products whose production is intimately linked to a specific landscape and territorial context. The use of landscape as a marketing tool, however, poses relevant operational problems related to communicating the landscape aesthetic visual quality to consumers who buy products in food stores and who have not visited the place of production.

In conclusion, our results suggest the need to conduct additional experiments aimed at further verifying the relationship between landscape preferences and food liking, and at identifying which communication strategies are most effective in using landscape aesthetic visual quality in the definition of food marketing strategies and the promotion of rural tourism.

## Figures and Tables

**Table 1:** Landscape stimuli classification according to Tveit et al. [23].

Juice	Landscape	Stimuli Classification
Orange	A	Negative disturbance effect:
		- presence of a harvesting machinery
	B	Positive ephemera * effect:
		- ripe oranges on the trees
Peach	A	Positive ephemera * effect:
		- flowering trees
	B	Negative naturalness effect:
		- absence of greenery
		Negative stewardship effect:
		- presence of branches deriving from pruning abandoned on the ground in bulk
Pear	A	Negative disturbance effect:
		- concrete poles that support the rows
	B	Positive ephemera * effect:
		- flowering trees
		Positive naturalness effect:
		- grass and flowers under the trees

* Tveit et al. [23] define ephemera as “elements and land-cover types changing with season and weather”.

**Table 2:** Study design.

Pair	Characteristics	Product 1 *	Product 2 *
Pair 1	Label:	A	B
	Taste:	orange	orange
	Expected landscape quality perception **:	low	high
Pair 2	Label:	A	B
	Taste:	peach	peach
	Expected landscape quality perception **:	high	low
Pair 3	Label:	A	B
	Taste:	pear	pear
	Expected landscape quality perception **:	low	high

* as described in the main text, Product 1 and Product 2 were the same juices. ** considering the stimuli provided (presented in Table 1) according to Tveit et al. [23].

**Table 3:** Effect of overall liking and landscape aesthetic visual quality on the probability that the participants are willing to pay more for one of the tasted juices.

*JUICE*_*PREF*		*LAND*_*PREF*			Total
		Not Preferred	Equally Preferred	Preferred	
	N	103	11	46	160
Not preferred	% within row	64.4	6.9	28.8	100.0
	% within column	58.2	36.7	26.0	41.7
	N	28	8	28	64
Equally preferred	% within row	43.8	12.5	43.8	100.0
	% within column	15.8	26.7	15.8	16.7
	N	46	11	103	160
Preferred	% within row	28.8	6.9	64.4	100.0
	% within column	26.0	36.7	58.2	41.7
	N	177	30	177	384
Total	% within row	46.1	7.8	46.1	100.0
	% within column	100.0	100.0	100.0	100.0

Pearson Chi squared = 46.39/*p* = 0.000.

**Table 4:** Factors influencing the probability that one juice is preferred over the other. Dependent variable: *HIGHEST*_*LIK* (dummy indicating the most preferred juice for each tasted pair).

Independent Variables	*β*	S.E.	z	*p*-Value
Landscape score	0.303	0.067	4.540	0.000
Constant	−1.954	0.379	−5.160	0.000

Chi squared = 23.96/*p* = 0.000. Loglikelihood = −249.501.

**Table 5:** Estimation of the probability that a juice was preferred (according to the overall liking score) over the other in the same pair as the landscape individual preferences score varies (probability expressed in percentage values).

	Landscape Aesthetic Visual Quality Rating						
	1	2	3	4	5	6	7
Probability that a juice is preferred over the other	16.9	20.61	26	32.23	39.17	46.57	54.12

**Table 6:** Willingness to pay more for one of the tasted juices (WTP) and preferred landscape (*LAND*_*PREF*).

WTP		*LAND*_*PREF*			Total
		Not Preferred	Equally Preferred	Preferred	
	N	82	9	39	130
willingness to pay less	% within row	63.1	6.9	30.0	100.0
	% within column	46.3	30.0	22.0	33.9
	N	56	12	56	124
willingness to pay equal	% within row	45.2	9.7	45.2	100.0
	% within column	31.6	40.0	31.6	32.3
	N	39	9	82	130
willingness to pay more	% within row	30.0	6.9	63.1	100.0
	% within column	22.0	30.0	46.3	33.9
	N	177	30	177	384
Total	% within row	46.1	7.8	46.1	100.0
	% within column	100.0	100.0	100.0	100.0

Pearson Chi squared = 31.74/*p* = 0.000.

**Table 7:** Landscape score and probability that the participants are willing to pay more for one of the tasted juices. Dependent variable: *HIGEST*_*WTP* (dummy indicating the highest WTP for each tasted pair).

Independent Variables	*β*	S.E.	z	*p*-Value
Landscape score	0.315	0.072	4.380	0.000
Constant	−2.372	0.416	−5.710	0.000

Chi squared = 19.20/*p* = 0.000. Loglikelihood = −235.002.

**Table 8:** Effect of the landscape aesthetic visual quality score on the probability that the participants are willing to pay more for one of the tasted juices (probability expressed in percentage values).

	Landscape Aesthetic Visual Quality Rating						
	1	2	3	4	5	6	7
Probability that the participants are willing to pay more	11.33	14.89	19.33	24.71	31.02	38.12	45.76

## Data Availability

The data that support the findings of this study are available on request from the corresponding author.

## Abbreviations

The following abbreviations are used in this manuscript:

*HIGHEST*_*LIK*	variable that assumes the value 1 for the most liked juice of each pair and 0 in the other cases (equal or lower overall liking)
*HIGEST*_*WTP*	variable that assumes the value 1 for the juice with the higher WTP of each pair and 0 in the other cases (equal or lower WTP)
*JUICE*_*PREF*	variable that assumes the value 3 for the more preferred juice of each pair, 2 when the juices received the same score and 1 for the less preferred
*LAND*_*PREF*	variable that assumes the value 3 for the more preferred landscape of each pair, 2 when the landscapes received the same score and 1 for the less preferred
WTP	Willingness To Pay
*WTP*_*PREF*	variable that assume the value 3 for the juice of each pair that received the higher WTP, 2 when the juices received the same WTP and 1 for the juice with the lower WTP

## Appendix A

**Table A1:** Participants socio-economic characteristics.

Variable	Levels	n	%	∑ %
Gender	Woman	26	40.6	40.6
	Man	38	59.4	100.0
	all	64	100.0	
Age Class	<25	25	39.1	39.1
	25–30	24	37.5	76.6
	>30	15	23.4	100.0
	all	64	100.0	
Area of Residence	Rural	38	59.4	59.4
	Urban	26	40.6	100.0
	all	64	100.0	
Education	Lower than Middle school diploma	1	1.6	1.6
	Middle school diploma	3	4.7	6.2
	High school diploma	33	51.6	57.8
	Bachelor or Master degree	27	42.2	100.0
	all	64	100.0	
Educational field	Not declared/General	3	4.7	4.7
	Agricultural sciences	26	40.6	45.3
	Biological sciences	10	15.6	60.9
	Humanities	17	26.6	87.5
	Architecture–Engineering	8	12.5	100.0
	all	64	100.0	

**Table A2:** Mean landscape rating about the aesthetic quality of the images representing the production area of the fruit used to prepare the juices.

Juice	Landscape	n	Mean Landscape Rating	Std. dev.
Orange *	Landscape A	64	4.05	1.28
	Landscape B	64	6.23	1.05
Peach *	Landscape A	64	6.48	0.87
	Landscape B	64	4.05	1.73
Pear *	Landscape A	64	4.22	1.69
	Landscape B	64	6.45	0.85

* The mean landscape scores are statistically different (*p* < 0.05).

**Table A3:** Overall liking rating for the tasted juices.

Juice	Label	n	Mean Overall Liking Rating	Std. dev.
Orange *	A	64	4.56	1.38
	B	64	5.05	1.47
Peach **	A	64	5.05	1.56
	B	64	5.16	1.52
Pear ***	A	64	4.91	1.56
	B	64	5.48	1.41

* The mean overall liking rating scores are statistically different (*p* < 0.1). ** The mean overall liking rating scores are not statistically different. *** The mean overall liking rating scores are statistically different (*p* < 0.05).

**Table A4:** Mean WTPs for the tasted juices.

Juice	Label	n	Mean WTP ^‡^	Std. dev.
Orange *	A	64	−3.52	10.72
	B	64	−0.08	11.43
Peach **	A	64	0.94	9.30
	B	64	−0.94	9.92
Pear ***	A	64	0.00	8.07
	B	64	3.52	8.76

^‡^ WTP expressed as percentage change with respect to the price paid for a juice in a supermarket. * The mean WTPs are statistically different (*p* < 0.1). ** The mean WTPs are not statistically different. *** The mean WTPs are statistically different (*p* < 0.05).
